# Sulfated Lactosyl Archaeol Archaeosome-Adjuvanted Vaccine Formulations Targeting Rabbit Hemorrhagic Disease Virus Are Immunogenic and Efficacious

**DOI:** 10.3390/vaccines11061043

**Published:** 2023-05-31

**Authors:** Bassel Akache, Andrew J. Read, Renu Dudani, Blair A. Harrison, Dean Williams, Lise Deschatelets, Yimei Jia, Vandana Chandan, Felicity C. Stark, Gerard Agbayani, Shawn R. Makinen, Usha D. Hemraz, Edmond Lam, Sophie Régnier, Wei Zou, Peter D. Kirkland, Michael J. McCluskie

**Affiliations:** 1 National Research Council Canada, Human Health Therapeutics, Ottawa, ON K1A 0R6, Canada; 2 Virology Laboratory, Elizabeth Macarthur Agricultural Institute, NSW Department of Primary Industries, Menangle, NSW 2567, Australia; 3 National Research Council Canada, Aquatic and Crop Resource Development, Montreal, QC H4P 2R2, Canada

**Keywords:** rabbit hemorrhagic disease virus, RHDV, sulfated lactosyl archaeol, SLA, vaccine, adjuvant, subunit, archaeosome

## Abstract

Vaccines play an important role in maintaining human and animal health worldwide. There is continued demand for effective and safe adjuvants capable of enhancing antigen-specific responses to a target pathogen. Rabbit hemorrhagic disease virus (RHDV) is a highly contagious calicivirus that often induces high mortality rates in rabbits. Herein, we evaluated the activity of an experimental sulfated lactosyl archaeol (SLA) archaeosome adjuvant when incorporated in subunit vaccine formulations targeting RHDV. The subunit antigens consisted of RHDV–CRM_197_ peptide conjugates or recombinant RHDV2 VP60. SLA was able to enhance antigen-specific antibody titers and cellular responses in mice and rabbits. Three weeks following immunization, antigen-specific antibody levels in rabbits vaccinated with RHDV2 VP60 + SLA were significantly higher than those immunized with antigen alone, with geomean titers of 7393 vs. 117. In addition, the SLA-adjuvanted VP60-based formulations were highly efficacious in a rabbit RHDV2 challenge model with up to 87.5% animals surviving the viral challenge. These findings demonstrate the potential utility of SLA adjuvants in veterinary applications and highlight its activity in different types of mammalian species.

## 1. Introduction

Subunit vaccines rely on the activity of adjuvants to enhance immunogenicity and generate sufficiently robust immune responses against the target pathogen to provide protection from disease. While a number of adjuvants have been approved for human or veterinary use [1,2], they may not always be suitable for new vaccines due to their activity profile (i.e., insufficient immunogenicity, induction of humoral vs. cellular responses, etc.). In addition, they may not be readily available due to supply or intellectual property issues. Indeed, the COVID-19 pandemic has reaffirmed the importance of vaccines and the need for a steady supply of effective and diverse adjuvants [3].

Archaeol-based liposomes (archaeosomes) contain lipids characterized by an archaea-specific structure where lipid tails consist of phytanyl side chains linked via an ether bond to the glycerol backbone [4]. Archaeosomes have been shown to be strong vaccine adjuvants in a number of preclinical models, greatly enhancing humoral and cellular antigen-specific immune responses [5,6]. Archaeosomes of sulfated lactosyl archaeol (SLA), generated semi-synthetically through the attachment of sulfated lactose to a biologically produced archaeol core, have been shown to robustly adjuvant the immunogenicity of a number of disease antigens, including hepatitis B surface antigen (HBsAg), hepatitis C E1/E2 glycoproteins, influenza hemagglutinin (HA), *Schistosoma mansoni* cathepsin B and SARS-CoV-2 spike in mice or hamsters [7,8,9,10,11,12]. As the formulation consists of a single lipid, SLA archaeosomes can be easily characterized and have been shown to be equally active with admixed antigen as with antigen encapsulated within the liposome [8,9,13]. They induce local cytokine/chemokine secretion, immune cell recruitment and antigen retention at the injection site [7,14,15]. Furthermore, SLA produced through a fully synthetic chemical process retain their adjuvant activity [16].

Vaccines are an important tool to maintain the health of companion animals or economically important livestock, with a large number of vaccines on the market targeting a wide range of diseases [1]. In addition, immunizing animals against zoonotic diseases could be an effective strategy to reduce transmission to humans [17]. Rabbit hemorrhagic disease virus (RHDV) is a highly contagious and lethal calicivirus that mainly infects *Oryctolagus cuniculus* rabbits [18]. While not a human pathogen, effective vaccines targeting RHDV are required, as it does pose a serious risk to the health of rabbits in the wild, those kept as companion animals or those used in industry. The RHDV genome consists of a positive-strand RNA encoding a number of nonstructural genes, as well as the VP60 capsid protein [19]. Classical RHDV, RHDV1, was identified in the 1980s and quickly spread across Europe and Asia [18]. In 2010, a new strain emerged, RHDV2, capable of infecting multiple hare species as well as *Oryctolagus cuniculus.* It has emerged as a serious threat, leading to outbreaks in many parts of the world.

Due to the largely conserved nature of the mammalian immune system, the technologies used in vaccines for animals are very similar to those used in humans. Vaccines targeting RHDV are generally of two types: (1) inactivated virus adjuvanted with aluminum salts or mineral oil (e.g., Filavac^®^ and Eravac) and (2) live viral vectored vaccine (e.g., Nobivac^®^ Myxo-RHD) where the RHDV capsid protein is expressed by a genetically modified myxoma virus [20,21]. While these vaccines have been highly effective against the original RHDV1 and more novel RHDV2 strains in areas with high disease burden, these vaccines have specific drawbacks. As the virus mainly replicates in rabbit liver cells which cannot be maintained in culture in vitro, the virus for the inactivated vaccines is routinely produced in live animals, which requires the use of more costly high containment laboratories to generate the material and then dispose of the contaminated tissues/waste. As there is a general risk of viral transmission/reversion with live viral vectored vaccines [22], their use for RHDV has been limited in certain regions. With an increased understanding of RHDV biology, the development of effective subunit vaccines that bypass these concerns is possible. Multiple studies have shown that the capsid protein, VP60, from RHDV1 or RHDV2 can self-assemble into genome-free virus-like particles (VLPs) that can induce protection at high antigen doses in a challenge model [23,24,25,26,27]. In addition, recent reports have identified a specific domain within RHDV1 VP60 that mediates cellular entry through interaction with nucleolin [28]. Immunization with a 17mer peptide corresponding to this VP60 domain mediated partial protection in a challenge model. However, to date, no marketed subunit vaccines for RHDV have been developed.

RHDV subunit vaccine formulations could be further optimized for clinical use through use of robust adjuvants such as SLA. As SLA has only been evaluated in rodents to date, it was not clear whether it would have similar immunostimulatory effects in rabbits. Herein, we evaluated the immunogenicity and efficacy of SLA-adjuvanted vaccine formulations targeting RHDV in mice and rabbits. Antigens consisted of self-assembled RHDV2 VP60 VLPs or RHDV peptides targeting the nucleolin interaction domain conjugated to cross-reactive material 197 (CRM_197_), a mutant nontoxic form of diphtheria toxin. CRM_197_ is a well-defined carrier protein, which has been used in multiple conjugated vaccines for a broad range of disease indications [29].

## 2. Materials and Methods

### 2.1. Vaccine Components

**Figure 1:**
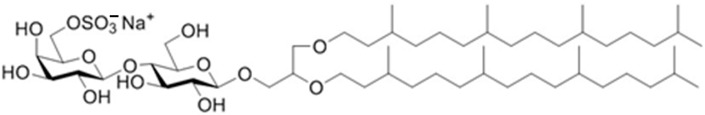
Chemical structure of SLA (6′-sulfate-β-d-Galp-(1,4)-β-d-Glcp-(1,1)-archaeol).

To produce the SLA adjuvant, total polar lipids were first extracted from *Halobacterium salinarum* (ATCC 33170) biomass, hydrolyzed and archaeol core purified as previously described [30]. Using the archaeol core, sulfated lactosyl archaeol (SLA; 6′-sulfate-β-d-Galp-(1,4)-β-d-Glcp-(1,1)-archaeol; Figure 1) was synthesized as reported previously [16,31]. Archaeosomes were formed by hydrating the SLA lipid in water as previously described [32]. SLA was diluted to the desired concentration in phosphate-buffered saline (PBS) and stored at 4 °C until use. SLA archaeosomes have been shown to be stable for up to 6 months when stored at 4 or 37 °C [33].

For the RHDV–CRM_197_ peptide conjugates, terminal N-acetylated peptides corresponding to the domain identified as responsible for nucleolin interaction with the RHDV1 capsid amino acids 468–484 [28] (Ac-RRTGDVNAAAGSTNGTQK) and the homologous region from RHDV2 (Ac-RRTGDINAEAGSTNGTQK) were synthesized by JPT Peptide Technologies GmbH (Berlin, Germany). The peptides were designed to allow conjugation to the CRM_197_ solely at the peptide’s C-terminal end. This was accomplished through addition of an N-terminal acetyl group and a C-terminal lysine residue which contains an amine sidechain. The peptides were dissolved in dimethyl sulfoxide (DMSO) and activated in PBS-based solution by 13 M disuccinimidyl suberate (DSS; ThermoFisher Scientific, Waltham, MA, USA). The reaction was monitored by high-pressure liquid chromatography (HPLC) using a C18 column with gradient 0.1% TFA-water/0.1% TFA-acetonitrile solvent system. After 6 h, the solution was directly injected onto a C18 column. Pure fractions were pooled and lyophilized. CRM_197_ (Fina Biosolutions, Rockville, MD, USA) solution was exchanged with conjugation buffer (0.1 M phosphate buffer, pH 6.8, 1.25% sucrose) using a 30K Amicon^®^ filter (Merck Millipore, Burlington, MA, USA). The activated peptide was dissolved in DMSO and added slowly to the CRM_197_ with stirring over 15 min. The reaction was monitored by HPLC (Agilent 1260 Infinity Analytical system, Santa Clara, CA, USA) using size exclusion chromatography (SEC; Superose 12, GE Healthcare, Chicago, IL, USA) and PBS as eluent. After 16 h, the conjugate was washed five times by adding 8 mL of 0.1 M phosphate buffer, pH 6.8, 1.25% sucrose and spinning through a 30K Amicon^®^ filter. The resulting solution was filter sterilized using a 0.22 µm filter and characterized by size exclusion (SE)-HPLC, matrix-assisted laser desorption/ionization (MALDI) and bicinchoninic acid assay. The conjugate was stored at −80 °C until further use. Solvents were obtained from Sigma-Aldrich (Burlington, MA, USA), Caledon Laboratory Chemicals (Georgetown, ON, Canada) or Fisher Scientific (Pittsburgh, PA, USA) and used without further purification.

The RHDV2 VP60 proteins were generated by GenScript (Piscataway, NJ, USA) using a Baculovirus expression system. The construct encoding the following amino acid sequence included a TEV protease recognition site (underlined) to enable cleavage of the 6x Histidine tag following protein purification: MHHHHHHENLYFQGEGKARAAPQGETAGTATTASVPGTTTDGMDPGVVATTSVVTTENASTSIATAGIGGPPQQVDQQETWRTNFYYNDVFTWSVADAPGNILYTVQHSPQNNPFTAVLSQMYAGWAGGMQFRFIVAGSGVFGGRLVAAVIPPGIEIGPGLEVRQFPHVVIDARSLEPVTITMPDLRPNMYHPTGNPGLVPTLVLSVYNNLINPFGGSTSAIQVTVETRPSEDFEFVMIRAPSSKTVDSISPADLLTTPVLTGVGTDNRWNGEIVGLQPVPGGFSTCNRHWNLNGSTFGWSSPRFAAIDHDGGNASFPGSSSSNVLELWYASAGSATDNPISQIAPDGFPDMSFVPFSGSTIPTAGWVGFGGIWNSNNGAPFVTTMQAYELGFATGAPSNPQPTTTTSGAQIVAKSIYGVANGINQTTAGLFVMASGVISTPNSSAITYTPQPNRIVNAPGTPAAAPIGKNTPIMFASVV**RRTGDINAEAGSTNGTQ**YGAGSQPLPVTVGLSLNNYSSALMPGQFFVWQLNFASGFMELGLSVDGYFYAGTGALATLIDLSELVDIRPVGPRPSTSTLVYNLGGTTNGFSYV. The nucleolin interaction domain is in bold.

Following removal of the tag, the purified protein was provided in a 50 mM Tris-HCl, 500 mM NaCl, 20% Glycerol, 1 mM DTT, pH 8.0 buffer. The aliquoted protein was stored at −80 °C until further use. Protein purity was confirmed to be >90% by SDS-PAGE. The absence of endotoxin contamination in all described antigens/adjuvants was confirmed using Endosafe cartridge-based Limulus amebocyte lysate tests (Charles River Laboratories, Charleston, SC, USA).

Transmission electron microscopy (TEM) was used to visualize the VLPs. TEM grids (formvar/carbon supported copper, 200 mesh, Sigma-Aldrich) were glow discharged prior to sample deposition. RHDV2 VP60 protein was applied to the surface of the grid and left for 30 s to allow for the sample to adsorb to the grid surface. Any extra sample was removed using filter paper, followed by washing two times with deionized water. Uranyl acetate (1%) was applied to the carbon surface of the grid, with excess solution removed using filter paper. Grids were left to air dry overnight before TEM imaging. Images were acquired on an FEI Titan3 80−300 TEM operated at 300 keV and equipped with a CEOS aberration corrector for the probe-forming lens and a monochromated field-emission gun.

### 2.2. Immunogenicity Studies in Mice and Rabbits

Female BALB/c mice and female New Zealand white rabbits were obtained from Charles River Laboratories (Saint-Constant, QC, Canada). Animals were housed at the animal facility of the National Research Council Canada (NRC) according to the guidelines of the Canadian Council on Animal Care. Antigen and preformed SLA archaeosomes were admixed and diluted in PBS on the day of immunization. Vaccine formulations were administered in a final volume of 50 or 500 µL per dose in mice or rabbits, respectively. In both species, the scruff of the neck was targeted for subcutaneous (s.c.) injections. Meanwhile, intramuscular injections (i.m.) were administered into the left cranial tibial muscle and left hind limb in mice and rabbits, respectively. Mice were bled via the submandibular vein for the collection of serum in serum separator tubes (Becton Dickinson, East Rutherford, NJ, USA), while blood in rabbits was collected in lithium-heparin-treated tubes (Becton Dickinson) via the central ear artery for the isolation of plasma and peripheral blood mononuclear cells (PBMCs) as described below. All rabbit procedures were conducted under slight sedation with acepromazine (Boehringer Ingelheim, Ingelheim, Germany), while mice were anesthetized with isoflurane (Baxter International, Deerfield, IL, USA) prior to immunizations. For the tolerability assessment, rabbits were monitored daily, with body weight and temperature recorded. For body temperature, transponders (Model IPTT-300, BioMedic Data Systems Inc., Seaford, DE, USA) were implanted subcutaneously prior to study start using a 12-gauge needle and the temperature was read in a noninvasive manner through the skin with a probe. In addition, local reactogenicity (i.e., erythema and edema) and clinical signs (i.e., lethargy, hunched posture, inappetence, body condition and fecal output) were monitored.

### 2.3. Antibody ELISA

Anti-RHDV2 VP60 IgG titers in serum or plasma were quantified by ELISA as previously described [34]. Briefly, 100 µL of 1 µg/mL RHDV2 protein (His-tagged version of protein described above) diluted in PBS was added per well of a 96-well high-binding ELISA plate (Thermo Fisher Scientific). Plates were coated overnight at room temperature (RT) and then washed five times with PBS/0.05% Tween20 (PBS-T; Sigma-Aldrich, St. Louis, MO, USA). To block the plates, 200 µL of 10% fetal bovine serum (FBS; Thermo Fisher Scientific) in PBS was added to each well and then plates were incubated for 1 h at 37 °C. After another round of washing as above, serially diluted samples in PBS-T with 10% FBS were added in 100 µL volumes and incubated for 1 h at 37 °C. After five washes with PBS-T (Sigma-Aldrich), 100 µL of goat anti-mouse IgG-HRP (Southern Biotech, Birmingham, AL, USA) or goat anti-rabbit IgG-HRP (Southern Biotech) was added at optimal dilution for 1 h at 37 °C. After a final set of washes with PBS-T, 100 µL/well of the substrate o-phenylenediamine dihydrochloride (OPD, Sigma-Aldrich) diluted in 0.05 M citrate buffer (pH 5.0) was added and plates kept in the dark. After 30 min, the reaction was stopped with 50 µL/well of 4N sulfuric acid (H_2_SO_4_). Plates were read spectrophotometrically at 450 nm. Titers for IgG in serum were defined as the dilution that resulted in an absorbance value (OD 450) of 0.2 and were calculated using XLfit software (ID Business Solutions, Guildford, UK). Samples that did not reach the target OD were assigned the value of the lowest tested dilution (i.e., 10 or 100) for analysis purposes.

For the peptide-specific ELISAs, plates were coated overnight at 4 °C with 10 µg/mL neutravidin (Thermo Fisher Scientific) diluted in PBS. Plates were washed three times with PBS alone, and then incubated for 2 h at room temperature with 1 µg/mL of biotinylated-RHDV1 or RHDV2 peptides diluted in PBS. Plates were then washed five times with PBS-T, blocked with 10% FBS and then treated the same as for the RHDV2 protein ELISA above.

### 2.4. ELISpot

The levels of CRM_197_- or RHDV2-specific T cells were quantified by ELISpot using a rabbit IFN-γ kit (Mabtech Inc., Cincinnati, OH, USA) as previously described [35]. PBMCs were isolated from 5 mL of rabbit blood by density gradient centrifugation. Blood was passed through a 70 µm cell strainer and overlaid on Histopaque^®^-1083 in 15 mL Accuspin™ tubes (Sigma-Aldrich) as per manufacturer’s recommendations. Blood was centrifuged for 30 min at 800× *g* at room temperature with the brake off. The buffy coat was collected and washed three times with PBS prior to resuspension in RPMI media (Thermo Fisher Scientific) containing 10% FBS (Thermo Fisher Scientific), 1% penicillin/streptomycin (Thermo Fisher Scientific), 1% glutamine (Thermo Fisher Scientific) and 55 µM 2-Mercaptoethanol (Thermo Fisher Scientific). Once PBMC yields were determined on a Cellometer (Nexcelom, Lawrence, MA, USA), cells were diluted to the appropriate concentration with the supplemented RPMI media. Whole protein (His-tagged RHDV2 or CRM_197_ as described above) was used to stimulate 4 × 10^5^ cells in duplicate at a final concentration of 5 µg/mL. Final volume per well was 200 µL. Cells were also incubated without any stimulants to measure background responses. Plates were incubated for approximately 20 h at 37 °C with 5% CO_2_, at which point the plates were washed and developed according to the manufacturer’s instructions. AEC substrate (Becton Dickinson) was used to visualize the spots. Spots were counted using an automated ELISpot plate reader (BioSys, Miami, FL, USA). For each animal, values obtained with media alone were subtracted from those obtained with the protein-stimulated cells to yield an overall number of antigen-specific IFN-γ^+^ spot-forming cells (SFCs)/10^6^ PBMCs per animal.

### 2.5. RHDV Challenge Studies in Rabbits

New Zealand white rabbits greater than 12 weeks of age were obtained and housed at the Elizabeth Macarthur Agricultural Institute. Animals were randomly assigned to 5 treatment groups consisting of 8 rabbits each, with 4 males and 4 females per group. They were confirmed to be free from detectable antibodies against RHDV1 and RHDV2. Animals were immunized s.c. at the scruff of the neck with vaccine formulations prepared as above. As a positive control, one group of animals received a commercial vaccine, Filavac^®^ VHD K C+V (Filavie, Sevremoine, France). On Days 14, 21 and 28 postimmunization, serum was collected from each animal to evaluate serological responses as determined by RHDV2-blocking ELISA [36]. Following blood collection on Day 28, rabbits were challenged orally with 0.5 mL of live RHDV2 virus suspension (EMAI strain M215; diluted in PBS, with each dose containing 1000 ID_50_ (infectious dose—50% end point)) [37]. Rabbits were housed individually in cages following challenge and monitored daily for clinical signs (e.g., lethargy, inappetence and seizures). All surviving rabbits were euthanized on Day 42 (14 days post-challenge). Liver samples were collected from each rabbit at time of death for testing for RHDV2 viral load by qRT-PCR. Total nucleic acid was extracted with the MagMAX-96 RNA Isolation Kit (Thermo Fisher Scientific) on a KingFisher™ 96 (Thermo Fisher Scientific) magnetic particle handling system following the manufacturer’s protocol. Nucleic acid was eluted in a 50 μL volume. The RHDV2 qRT-PCR assay utilized the AgPath-IDTM One-Step RT-PCR kit (Thermo Fisher Scientific). A total of 5 μL of purified nucleic acid was added to a 20 μL reaction mix. Primers and the probe [38] were added and samples run in an ABI 7500 Fast thermal cycler (Applied Biosystems, Waltham, MA, USA) according to the master mix manufacturer’s standard parameters. Results were expressed in terms of the cycle number at which the sample fluorescence plot crossed the 0.05 threshold (the Ct value). Collected sera were tested for antibodies directed towards RHDV2 by an anti-RHDV blocking assay similar to that previously described [36,37]. The ELISA plates were first coated with a polyclonal anti-RHDV2 rabbit antiserum as the capture antibody, followed by a semi-purified RHDV2 antigen (both anti-sera and antigen were produced at EMAI). The test serum was then added to the plate and, after an incubation period of 30 min, a peroxidase-conjugated RHDV2-specific monoclonal antibody (4H12, supplied by IZSLER, Brescia, Italy) was added to measure the level of blocking of the monoclonal antibody by the test serum. Results were calculated as a percentage inhibition (PI), with a cut-off point for a positive result set at >40%.

### 2.6. Statistical Analysis

GraphPad Prism^®^ version 9 (GraphPad Software, San Diego, CA, USA) was used to analyze the data. Statistical significance of the difference between groups was calculated by: (1) for 2 groups, unpaired two-tailed Student’s *t*-test or (2) for 3 or more groups, one-way analysis of variance (ANOVA) followed by post hoc analysis using Tukey’s (comparison across all groups) multiple comparison test. Antibody and ELISpot data were log transformed prior to statistical analysis. Survival curve analyses were carried out using the Gehan–Breslow–Wilcoxon method. For all analyses, differences were considered to be not significant with *p* > 0.05. Significance was indicated in the graphs as follows: * *p* < 0.05, ** *p* < 0.01, *** *p* < 0.001 and **** *p* < 0.0001.

## 3. Results

### 3.1. RHDV–CRM_197_ Peptide Conjugates

Based on recent reports demonstrating the protective potential of free peptide-based vaccines targeting a specific domain within VP60 responsible for cellular internalization of the RHDV1 [28], we sought to generate vaccine antigens with enhanced immunogenicity to the peptide domains through generation of RHDV peptide CRM_197_ conjugates. As RHDV2 has largely supplanted RHDV1 as the predominant viral strain in most parts of the world, we sought to generate a bivalent vaccine formulation by separately conjugating peptides corresponding to homologous regions of both viruses, which are highlighted (in bold) by two amino acid changes in the target region: RHDV1_468-484_: RRTGDVNAAAGSTNGTQ vs. RHDV2_468-484_: RRTGDINAEAGSTNGTQ. HPLC-SEC and MALDI analysis confirmed successful peptide loading on the conjugates with a demonstrable increase in average molecular weight with approximately three peptides conjugated per carrier protein (Table 1).

**Figure 2:**
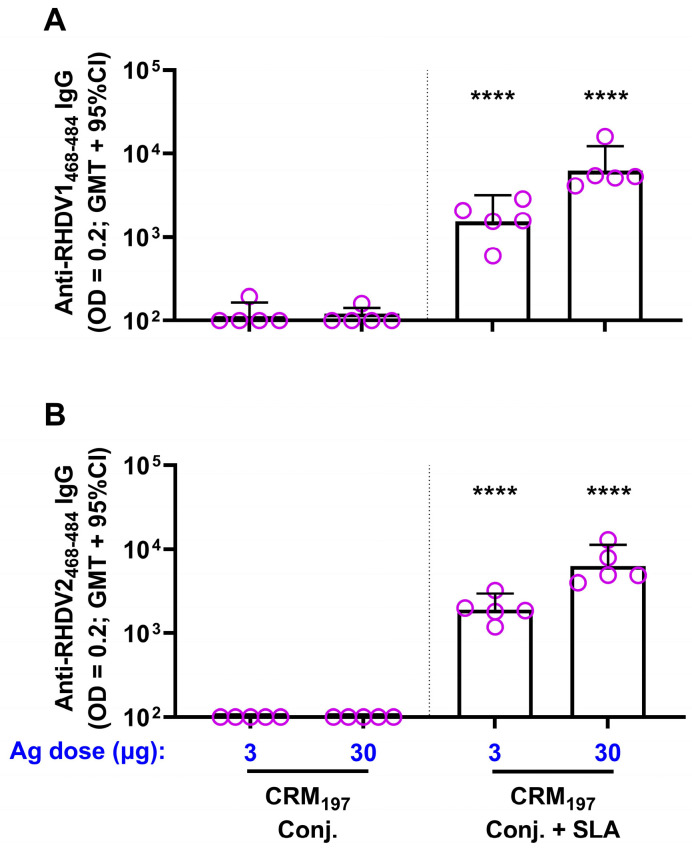
Immunogenicity of SLA-adjuvanted CRM_197_–RHDV vaccine formulations in mice. BALB/c mice (*n* = 5/group) were immunized i.m. with 3 or 30 µg of CRM_197_–RHDV1 (**A**) or RHDV2 (**B**) conjugates alone or adjuvanted with SLA (1 mg) on Day 0. Serum was collected on Day 26 and analyzed by ELISA to determine the levels of antigen-specific antibody titers against the corresponding biotinylated peptides. Grouped data is presented as geometric mean + 95% confidence interval (CI). Statistical significance of differences for the adjuvanted vs. unadjuvanted groups with equivalent antigen dose is shown: ****: *p* < 0.0001 by one-way ANOVA followed by Tukey’s multiple comparisons test.

The immunogenicity of each of the conjugates was then confirmed in mice. While a single vaccination with 3 or 30 µg of each conjugate alone generated weak antibody titers (geomean titers (GMT) of 100–114) to the corresponding peptide sequence, inclusion of SLA significantly enhanced antibody titers, with GMT of ~2000 and ~8000 with 3 and 30 µg of either antigen, respectively (Figure 2). A strong antigen dose sparing effect was seen with the inclusion of SLA, as antibody titers obtained with 3 µg of either SLA-adjuvanted conjugate were >14-fold higher than a 10-fold higher dose (30 µg) of the corresponding unadjuvanted conjugate.

**Figure 3:**
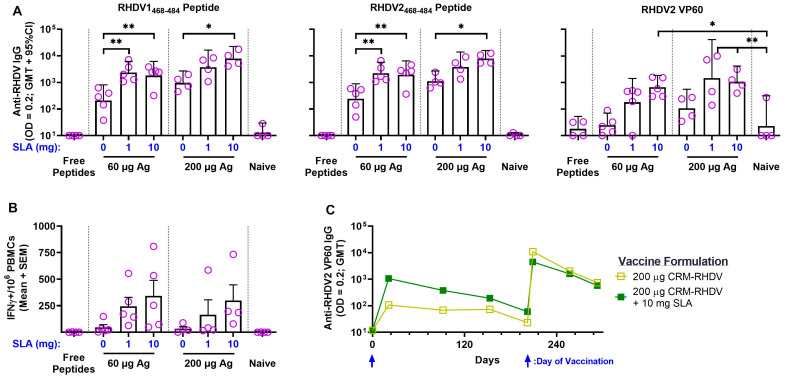
Immunogenicity of SLA-adjuvanted CRM_197_–RHDV vaccine formulations in rabbits. New Zealand white rabbits (*n* = 4–5/group) were immunized i.m. with formulations consisting of various doses of CRM_197_–RHDV1 and RHDV2 conjugates alone or adjuvanted with SLA (1 vs. 10 mg) on Day 0. Control groups included rabbits immunized with unconjugated free peptides (1 mg each of RHDV1_468–484_ and RHDV2_468–484_) and naïve unvaccinated animals. (**A**) Plasma was collected on Day 21 and analyzed by antibody ELISA to determine the levels of antigen-specific antibody titers against each of the biotinylated RHDV peptides (left panel: RHDV1 and middle panel: RHDV2) or RHDV2 VP60 capsid protein (right panel). Grouped data are presented as geometric mean + 95% confidence interval (CI). (**B**) PBMCs were isolated from blood collected on Day 7 and analyzed by IFN-γ ELISpot to determine the levels of CRM_197_-specific T cells. Grouped data are presented as mean + standard error of mean (SEM). (**C**) The longevity of immune responses against RHDV2 VP60 capsid protein in rabbits immunized with 200 µg of CRM_197_–RHDV alone or adjuvanted with SLA (10 mg) was assessed by antibody ELISA in serum collected at various time points. Animals received a 2nd vaccine dose on Day 202. Grouped data are presented as geometric mean. Statistical significance of differences between the indicated groups is shown: *: *p* < 0.05 and **: *p* < 0.01 by one-way ANOVA followed by Tukey’s multiple comparisons test.

Having confirmed the immunogenicity of the conjugate antigens in vivo, we next immunized rabbits with bivalent formulations of the CRM_197_–RHDV conjugates. The rabbits received a total dose of 60 or 200 µg of the conjugates, with half of each dose consisting of CRM_197_–RHDV1 or CRM_197_–RHDV2, alone or adjuvanted with SLA. As this was the first time SLA was administered in rabbits, we evaluated two different SLA doses: 1 mg (the standard dose used in mice) and 10 mg. As controls, groups of naïve unimmunized rabbits, as well as animals immunized with free peptides (i.e., unconjugated) were included. No to low antigen-specific titers were seen in rabbits immunized with the unconjugated peptides or in the naïve controls (Figure 3A). In contrast to what was seen in mice, the CRM_197_–RHDV conjugates without adjuvant were able to induce peptide-specific IgG titers that were significantly higher than seen in the naïve rabbits (*p* < 0.001). The inclusion of adjuvant further enhanced antibody titers against both RHDV1 and RHDV peptides. For example, 60 µg of CRM_197_–RHDV alone induced GMT of 208 against RHDV1_468–484_, while inclusion of 1 and 10 mg of SLA induced GMT of 2379 and 1826, respectively (*p* < 0.01). As was seen in mice, an antigen-sparing effect was observed. Addition of 1 or 10 mg SLA to 60 µg of antigen induced higher antibody titers than those obtained following immunization with 200 µg of unadjuvanted conjugate. In addition, the reactivity against whole recombinant RHDV2 VP60 capsid protein was also evaluated by indirect ELISA. Indeed, the anti-RHDV peptide antibodies were able to cross-react against the whole protein, with significantly higher titers vs. the naïve controls seen in rabbits immunized with most of the adjuvanted formulations, i.e., 60 µg CRM_197_–RHDV + 10 mg SLA and 200 µg CRM_197_–RHDV + 1 or 10 mg SLA.

As anti-carrier CD4^+^ T cells are known to enhance the quality and magnitude of the immune response to the conjugated antigenic moieties through the direct activation of B cells that have internalized the carrier antigen complex [39], CRM_197_-specific T cells were quantified in PBMCs collected Day 7 postimmunization. While 60 or 200 µg of the RHDV conjugates alone were able to induce an average of <50 IFN-γ^+^ SFCs/10^6^ PBMCs, the inclusion of SLA at either antigen/adjuvant dose level induced an average of 165–343 IFN-γ^+^ SFC/10^6^ PBMCs (Figure 3B). This did not reach a level of statistical significance at the group level due to the general variability in responses and low number of animals per group. However, if the values from the various groups were combined into unadjuvanted vs. adjuvanted conjugate categories, significantly different average values of 39 vs. 266 IFN-γ^+^ SFCs/10^6^ PBMCs were seen, respectively (*p* < 0.001 by Student’s t-test). Finally, the longevity of the immune responses in two groups (200 µg of CRM_197_–RHDV ± 10 mg SLA) was measured over 6 months postvaccination (Figure 3C). The levels of IgG antibodies reactive to RHDV2 VP60 decreased gradually over time, declining from a GMT of 1066 at Day 21 to 60 at Day 202 in the 200 µg of CRM_197_–RHDV + 10 mg SLA group. Administration of a second vaccine dose on Day 202 boosted the antibody levels to GMT >4000 7 days later, but a decline in titers over time was seen again with the titers measuring <750 in both groups at Day 293 (~3 months following boost). Interestingly, the adjuvant effect was no longer evident between these two groups following boost, as GMT were generally similar in both groups.

**Figure 4:**
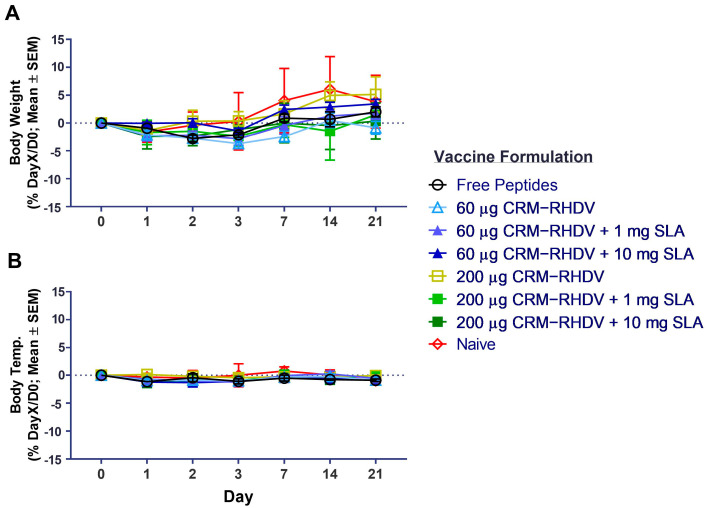
Tolerability of SLA-adjuvanted CRM_197_–RHDV vaccine formulations in rabbits. Vaccinated rabbits described above in Figure 2 were monitored for changes in body weight (**A**) and temperature (**B**) up to 21 days following vaccination. Grouped data are presented as mean ± standard error of mean (SEM).

As safety and tolerability are important characteristics of vaccine adjuvants, we monitored changes in body weight and temperature following the first vaccine dose in the animals in this study. Importantly, no significant changes were seen between the naïve and immunized rabbits over the 3 weeks following immunization, indicating the vaccine components were generally well tolerated (Figure 4). In addition, no treatment-related clinical signs or local reactogenicity were observed in any of the animals.

### 3.2. RHDV2 VLPs

**Figure 5:**
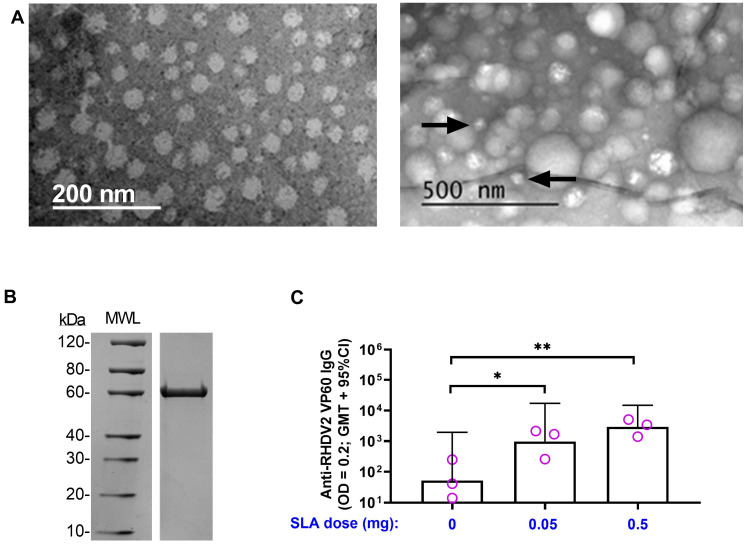
Immunogenicity of SLA-adjuvanted RHDV2 VLP vaccine formulations in mice. (**A**) Purified recombinant RHDV2 VP60 protein was visualized by transmission electron microscopy (TEM) to assess its ability to form virus-like particles (VLPs). The protein was imaged either alone (left panel) or when combined with SLA (1:33 *w*/*w*). Black arrows indicate particles with size corresponding to RHDV2 VLPs. (**B**) RHDV2 VP60 protein was analyzed under reducing conditions by SDS-PAGE and size compared to molecular weight ladder (MWL). (**C**) BALB/c mice (*n* = 3/group) were immunized s.c. with 1.5 µg of RHDV2 VLP alone or adjuvanted with SLA (0.05 or 0.5 mg) on Day 0. Serum was collected on Day 20 and analyzed by antibody ELISA to determine the levels of antigen-specific antibody titers against the RHDV2 capsid protein. Grouped data are presented as geometric mean + 95% confidence interval (CI). Statistical significance of differences between the indicated groups is shown: *: *p* < 0.05 and **: *p* < 0.01 by one-way ANOVA followed by Tukey’s multiple comparisons test.

Although the RHDV–CRM conjugate vaccine formulations were able to induce strong humoral and cellular antigen-specific responses when adjuvanted with SLA, the waning of responses over time was not optimal. Therefore, we next sought to evaluate the ability of SLA to enhance anti-RHDV responses when administered with an alternative form of antigen, namely RHDV2 VP60 protein. Recombinant protein was produced in a baculovirus expression system and, once purified, size and purity were confirmed by SDS-PAGE (Figure 5B). It was also tested for its ability to form VLPs by electron microscopy (either alone or when combined SLA). An inability to form VLPs could impact its immunogenicity profile and thereby increase the antigen doses required for the induction of antigen-specific immune responses. When visualized alone, VLPs of ~30 nm were clearly detected with a morphology similar to those previously reported by other groups (Figure 5A) [23]. When combined with SLA (at an average size of ~100 nm and 33-fold higher concentration of SLA to RHDV2 VP60 protein), most of the observed particles corresponded to what we would expect for SLA. Some smaller ~30 nm particles were still observed, but it is hard to conclude definitely that they are RHDV VLPs. To confirm the immunogenicity and compatibility of RHDV VLPs with SLA, a small pilot study was conducted in mice. Mice were immunized s.c. with 1.5 µg of Ag alone or with 0.05 or 0.5 mg of SLA. While SLA-adjuvanted formulations are usually given i.m., s.c. administration here allowed us to confirm SLA activity via this route, which is generally preferred for clinical administration of vaccines to rabbits. Indeed, antigen-specific antibodies were detectable in all mice, with inclusion of SLA at either dose leading to a significant increase in anti-RHDV2 VP60 IgG titers vs. antigen alone (*p* < 0.05; Figure 5C).

**Figure 6:**
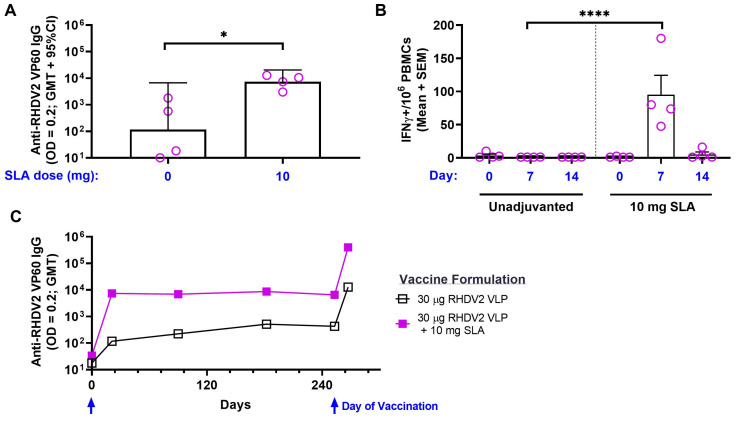
Immunogenicity of SLA-adjuvanted RHDV2 VLP vaccine formulations in rabbits. (**A**) New Zealand white rabbits (*n* = 4/group) were immunized s.c. with formulations consisting of 30 µg RHDV2 VLP alone or adjuvanted with SLA (10 mg) on Day 0. Plasma was collected on Day 21 and analyzed by antibody ELISA to determine the levels of antigen-specific antibody titers against the RHDV2 capsid protein. Grouped data are presented as geometric mean + 95% confidence interval. (**B**) PBMCs were isolated from blood collected on Days 0 (pre-vaccination), 7 and 14 and analyzed by IFN-γ ELISpot to determine the levels of RHDV-specific T cells. Grouped data are presented as mean + standard error of mean. (**C**) The longevity of immune responses in rabbits was assessed by antibody ELISA in serum collected at various time points. Animals received a 2nd vaccine dose on Day 253. Grouped data are presented as geometric mean + 95% confidence interval. Statistical significance of differences between the indicated groups is shown: *: *p <* 0.05 and ****: *p* < 0.0001 by unpaired two-tailed Student’s *t*-test.

Rabbits were immunized s.c. with 30 µg of RHDV2 VLPs alone or with 10 mg of SLA. At Day 21, RHDV2-specific antibodies were detectable in immunized rabbits with antigen + SLA inducing significantly higher GMT of 7393 vs. 117 with antigen alone (*p* < 0.05; Figure 6A). SLA was also able to induce RHDV2-specific T cells, with an average of 95 IFN-γ^+^ SFCs/10^6^ PBMCs seen at Day 7, which was significantly higher than the average of 1 IFN-γ^+^ SFC/10^6^ PBMCs seen with rabbits immunized with antigen alone at a similar time point (*p* < 0.0001; Figure 6B). Meanwhile, no to low levels of T cells were seen in both groups prior to vaccination at Day 0 and at Day 14. Finally, the longevity of the antigen-specific antibody responses was tracked in these animals over time. The GMT were well maintained in the immunized rabbits for up to 253 days, with GMT of 6514 at Day 253 vs. 7393 at Day 21 in rabbits administered RHDV2 VLPs + SLA (Figure 6C). The antigen-specific immune responses were amenable to boosting, with a second vaccine dose on Day 253 inducing >50-fold increase in anti-RHDV2 VP60 IgG titers with GMT of 399,987 seen 14 days later in rabbits immunized with the adjuvanted formulation. This was significantly higher than the GMT of 12,769 (*p* < 0.05) detected in rabbits immunized with antigen alone at the same time point.

### 3.3. Efficacy of SLA-Adjuvanted Vaccine Formulations in RHDV2 Challenge Model

**Figure 7:**
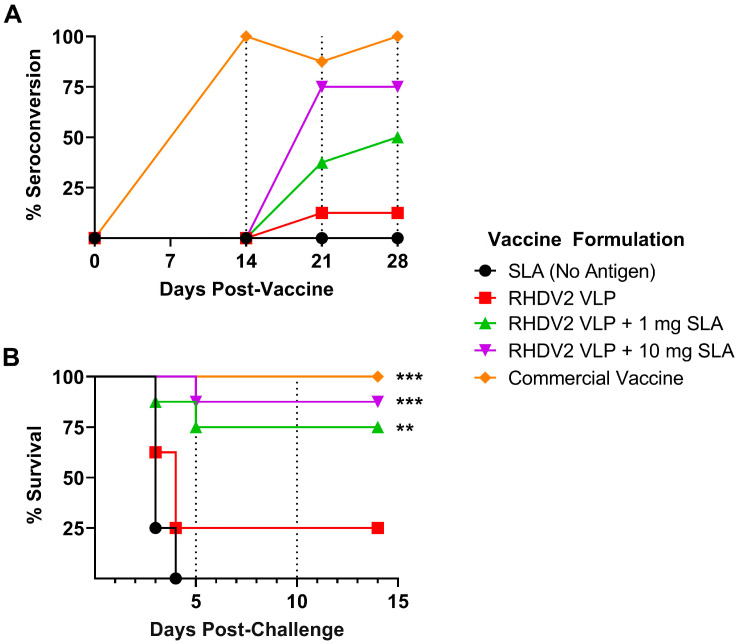
Efficacy of SLA-adjuvanted RHDV2 VLP vaccine formulations in RHDV2 challenge model. New Zealand white rabbits (*n* = 8/group) were immunized s.c. with formulations consisting of 30 µg RHDV2 VLP alone or adjuvanted with SLA (1 or 10 mg) on Day 0. Control groups included rabbits immunized with 10 mg SLA alone without antigen or a commercial RHDV vaccine. (**A**) Serum was collected on Days 14, 21 and 28 for analysis by RHDV2 ELISA blocking assay to determine the seroconversion rate per group. (**B**) On Day 28, rabbits were challenged orally with live RHDV2 virus and assessed daily for clinical signs. Animals were euthanized if they met a humane endpoint or at 14 days post viral challenge. Percent survival over time is shown. For survival, statistical significance of differences between the indicated groups vs. the SLA (no antigen) control is shown: **: *p <* 0.01 and ***: *p <* 0.001 by Gehan–Breslow–Wilcoxon method.

Based on the strong sustained immune responses observed with the RHDV2 VLP + SLA vaccine formulations, they were then evaluated in an RHDV2 viral challenge model. Again, rabbits were immunized with 30 µg RHDV2 VLP alone or adjuvanted with SLA. As a dose of 1 mg SLA was active with CRM_197_–RHDV conjugate antigens in rabbits (Figure 3), formulations containing either 1 or 10 mg SLA were tested here to determine if 1 mg SLA was sufficient to mediate protection. To control for potential immunostimulatory properties of the adjuvant alone on the innate immune system, rabbits in the negative control group were administered 10 mg of SLA alone without any antigen. A positive control group was immunized with a commercial inactivated viral vaccine. Serum was collected at various time points pre- and postimmunization for assessment of the serological response as determined by an anti-RHDV2 blocking assay. By Day 28 postimmunization, all animals in the positive control group had seroconverted, while 50 and 75% seroconversion was seen in animals immunized with 30 µg antigen adjuvanted with 1 and 10 mg of SLA, respectively (Figure 7A). The groups receiving antigen or adjuvant alone showed ≤12.5% seroconversion at all time points tested. Interestingly, the kinetics of seroconversion appear different between the groups, with slower induction of blocking antibodies seen with the recombinant protein-based formulations than with the commercial vaccine: 100% seroconversion with the commercial vaccine vs. 0% in all other groups at Day 14.

All rabbits were challenged on Day 28 with live RHDV2 and monitored for their ability to resist infection over 2 weeks. As expected, all animals in the negative control adjuvant-alone group succumbed to infection by Day 4 (Figure 7B). Rabbits immunized with antigen alone had a low level of protection, with 25% of animals surviving challenge until the study’s endpoint, but this was not significantly different from the negative control. Inclusion of SLA induced a significant increase in survival (*p <* 0.01), with 75 to 87.5% survival seen in animals immunized with RHDV2 VLPs adjuvanted with 1 and 10 mg SLA, respectively. While 100% survival was seen with the commercial vaccine, this was not statistically different to the SLA-adjuvanted formulations. Finally, analysis of liver samples collected at the time of euthanasia confirmed the presence of high levels of virus (i.e., Ct values of 6.8–11) in all animals that succumbed to infection between Days 3 and 5 post-challenge (Table 2). Most animals that survived challenge and were euthanized at study endpoint at Day 14 post-challenge had no detectable viral genome. However, 3 of the 13 surviving animals in the RHDV2 VLP + SLA groups had detectable but lower viral loads, with Ct values of 21.8–33.1.

## 4. Discussion

SLA’s adjuvant effects were first established in rodent species such as mice. In this study, we have demonstrated the activity and utility of the SLA adjuvant system in a larger mammalian species, rabbits. SLA was able to augment antigen-specific antibodies and T cells in rabbits to two different types of antigens: a carrier-linked peptide vaccine and a recombinant protein VLP. In the case of the RHDV2 VLPs, SLA was able to induce functional responses that were efficacious in protecting rabbits from disease in a challenge model.

The use of recombinant VP60-based vaccines targeting RHDV1 or RHDV2 have been evaluated previously. In studies measuring responses after a single vaccine dose, the amount of purified antigen included in the formulations varied greatly, ranging from 500 to 5000 µg per dose [23,27,40]. While most of these did not incorporate an adjuvant, one did include a water-in-oil-in-water adjuvant Montanide™ ISA 201 VG [40]. One of the first studies to attempt this recombinant VP60-based vaccine approach formulated 30 µg of antigen with water-in-oil adjuvant Montanide™ ISA 50 but utilized a multiple-vaccination strategy (two vaccine doses, 14 days apart) to demonstrate survival from viral challenge with RHDV1 [24]. In our study, we clearly demonstrated that inclusion of SLA in the vaccine formulation allowed for up to 88% survival following a single immunization with a vaccine containing only 30 µg of antigen. While we did not achieve 100% efficacy in our stringent challenge model, it is conceivable that this could be achieved with the use of slightly higher antigen doses or following a booster dose. This would be of interest to evaluate in future studies. The amount of antigen dose sparing discussed above would have important implications on the cost of the formulation. The cost of including the adjuvant would also need to be taken into account when designing the vaccine formulation. The SLA generated for this study was made through a semi-synthetic process where the sulfated lactose domain is chemically conjugated to biologically derived archaeol, which has been purified from the biomass of the aerobically cultured archaeal species *H. salinarum*. There is also the option of manufacturing a purely synthetic SLA, generated chemically without any archaeal precursors, as it was shown to be equally active in vivo [16]. We are currently optimizing and adapting the manufacturing process to generate SLA cost-effectively at a larger scale suitable for clinical applications. This work will better inform us on the ultimate cost of generating SLA for commercial applications.

In the challenge study, the efficacy of our SLA-adjuvanted RHDV2 VLP formulations was compared to a commercially available RHDV vaccine. The rates of survival induced by these formulations were statistically similar, but subtle differences in the kinetics and degree of seroconversion were observed. The commercial vaccine’s antigen component consisted of homogenized liver extract from RHDV-infected rabbits which had been treated to inactivate the virus. To enhance its immunogenicity, the liver extract was paired with an aluminum salt-based adjuvant. It should also be noted that the commercial vaccine contained antigens from both RHDV1 and RHDV2 genotypes. The amount of virus included in each dose is unclear. As the viral antigen is generated in rabbits, it is possible that it contains certain features (e.g., glycosylation pattern) that would more closely mimic the capsid protein found on the viral pathogen than the insect cell-derived antigen used in the SLA-adjuvanted formulations. Therefore, differences in antigen type, format and quantity, as well as the adjuvant used, could explain the minor differences seen between the commercial and experimental formulations tested above. In future studies, it would be informative to compare the physical characteristics and immunogenicity (when combined with SLA) of a mammalian vs. insect cell-produced RHDV VP60 antigen. In addition, it would be interesting to confirm the efficacy of SLA-adjuvanted bivalent formulations including a combination of RHDV1- and RHDV2-based VLPs against both viruses.

In this study, we were also able to confirm the high tolerability profile of SLA in another mammalian species. Previous studies in mice had shown that it was safe and well tolerated at doses of up to 10 mg [14]. Studies conducted under Good Laboratory Practice (GLP) standards in rats have also shown SLA to have a favorable safety profile when administered alone at doses of up to 10 mg (McCluskie et al., unpublished data). As safety and tolerability of a vaccine formulation may also depend on the specific antigen, confirmatory studies will need to be repeated once the vaccine doses/formulations are established for any clinical formulation. Interestingly, we were able to utilize similar SLA dose levels of 1 to 10 mg in rabbits with average body weights >4 kg compared to mice weighing >100 times less. While responses tended to be slightly better with 10 vs. 1 mg in the rabbit challenge model, we found generally similar activity with both doses of adjuvant when coupled with the CRM_197_–RHDV conjugates (Figure 3). While further study is required, these results indicate that much higher doses of SLA may not be required when moving to even larger animal species or humans. This has been seen with other adjuvant types, such as emulsion or aluminum salt-based adjuvants, where adjuvant concentration may be more relevant than a dose based on the size/weight of the immunized subject [41,42]. We were also able to demonstrate that SLA retains its adjuvant activity when administered s.c. Almost all studies conducted to date with SLA have been administered i.m. to mimic the favored immunization route for parenteral vaccines in humans, but data above (Figure 5, Figure 6 and Figure 7) suggest that s.c. administration could be a viable option with SLA-adjuvanted vaccines in the future. This could have important implications for multiple veterinary applications where s.c. is the preferred vaccination route.

SLA was also able to effectively enhance antibody responses to the RHDV peptides encompassing the nucleolin interaction domain. Zhu et al. [28] have shown that immunization with unconjugated peptide did induce RHDV-specific antibody responses and some protection from RHDV1 challenge. We found that free peptides were weakly immunogenic with limited measurable induction of antigen-specific antibodies. This may be due to slight differences in antigen sequence or structure (presence of N- and C-terminal acetyl group and lysine, respectively) or the purity of the peptides. In our study, the conjugate vaccines did generate significantly higher levels of peptide-specific and RHDV2 VLP-reactive antibodies than the free peptides. In future studies, it would be interesting to evaluate if they would be protective against viral challenge, especially for RHDV2, which has not yet been shown to rely on nucleolin for cellular entry. However, the waning of responses over time in our rabbit study could indicate that some optimization of the RHDV conjugates may be required. It would be interesting to determine if altered peptide loading per CRM_197_ molecule or the masking of terminal neo-epitopes on the linear peptides would enhance longevity of responses [43]. An altered peptide loading profile on the carrier could potentially reduce the induction of anti-carrier antibodies, which have been previously shown to reduce the levels of responses following subsequent vaccinations with conjugate antigens using the same carrier [44]. While CRM_197_ IgG titers were not measured in our study, potential induction of higher levels of anti-CRM_197_ antibodies with the SLA-adjuvanted formulation could have impacted the vaccine immunogenicity following the second immunization, leading to a similar level of responses with the unadjuvanted vs. SLA-adjuvanted RHDV–CRM conjugates following boost.

In summary, our data demonstrate the strong adjuvant activity of SLA in larger mammalian species such as rabbits and its potential utility in a recombinant protein-based vaccine targeting RHDV. Our formulation could serve as the basis for a novel safe RHDV vaccine that circumvents some of the safety and manufacturing concerns associated with formulations currently on the market. As such, we believe that SLA in general and these RHDV vaccine formulations in particular warrant further development as novel vaccination technologies.

## Figures and Tables

**Table 1:** Characterization of CRM_197_–RHDV1 and RHDV2 conjugates.

CRM_197_ Conjugate	Weight (Da)	Molar Ratio	Peptide %
Untagged	58,511	N/A	0
CRM_197_–RHDV1	64,551	3.04	9.3
CRM_197_–RHDV2	64,675	3	9.5

**Table 2:** Ct values indicating viral load in the liver at the time of euthanasia.

	Vaccine Formulation				
Animal	SLA	RHDV2 VLP	RHDV2 VLP + 1 mg SLA	RHDV2 VLP + 10 mg SLA	Commercial Vaccine
1	**8.4**	ND	ND	ND	ND
2	**10.5**	ND	ND	ND	ND
3	**10.2**	**11**	ND	ND	ND
4	**9.7**	**10.2**	ND	ND	ND
5	**9.1**	**9.8**	33.1	ND	ND
6	**7**	**9.2**	25.1	ND	ND
7	**6.8**	**8.6**	**9.9**	21.8	ND
8	**6.8**	**8.5**	**9.1**	**9**	ND

ND: non-detectable. Numbers in bold indicate animal was found dead or euthanized prior to end of study on Day 42.

## Data Availability

The data presented in this study are available on request from the corresponding author. The data are not publicly available due to privacy concerns.
